# Parasites and Microbiota: Dual Interactions and Therapeutic Perspectives

**DOI:** 10.3390/microorganisms12102076

**Published:** 2024-10-16

**Authors:** Hayat S. Al-Rashidi, Eman S. El-Wakil

**Affiliations:** 1 Department of Biology, College of Science, Qassim University, Buraydah 52571, Saudi Arabia; h.alreshidi@qu.edu.sa; 2 Department of Parasitology, Theodor Bilharz Research Institute, Kornaish El-Nile, Warrak El-Hadar, Imbaba, P.O. Box 30, Giza 12411, Egypt

**Keywords:** parasites, microbiota, host pathogen interaction, dysbiosis, immunity, probiotics

## Abstract

The human gut hosts a diverse and active community of bacteria that symbiotically support the physiology, metabolism, and immunity of the intestinal lining. Nevertheless, a dynamic community of parasites (helminths and protozoa) may share a habitat with gut-dwelling microbiota. Both microbiota and parasites can significantly change the physical and immunological environment of the gut, thus generating several mechanisms of interaction. Studying this field is crucial for understanding the pathogenesis of parasitic diseases. Additionally, intestinal microbiota and gut-dwelling parasites may interact with each other and with the host immunity to alleviate or exacerbate the disease. These interactions can alter the pathogenicity of both parasites and microbiota, thereby changing the infection outcomes and the overall disease profile. Parasites and microbiota interactions occur via several mechanisms, including physical alteration in both the gastrointestinal microenvironment and the adaptive and innate immune responses. By modulating the microbiota, treating parasitic infections and microbiota dysbiosis may be improved through knowing the mechanisms and consequences of the interactions between intestinal parasites and the microbiota. Thus, new biological tools of treatment including probiotics can be introduced, particularly with the emergence of drug resistance and adverse effects.

## 1. Introduction

### 1.1. Pathogens and Microbiota

Pathogens are often seen as foreign invaders targeting our bodies, but they are primarily trying to survive and spread. There are three primary types of invading pathogens: bacteria, viruses, and parasites (which include helminths and protozoa). Pathogens are dependent on their hosts, and in a parasitic relationship, one organism usually thrives and multiplies at the other’s expense [1,2].

Microbiota comprises commensal microbes that live within the human body in a symbiotic relationship. The term “microbiota” comprises two syllables: “micro” denotes its microscopic size, and “biota” refers to the bacterial ecology that resides at a particular site within the host [3].

Typically, pathogens are different from the microbiota. Normal microbiota become problematic only when they enter normally sterile areas of the body, such as during a bowel rupture that allows gut flora into the peritoneal cavity, causing peritonitis, or when the immune system is compromised. Dedicated pathogens, on the other hand, can create extremely specialized mechanisms that allow them to get past biochemical and cellular barriers and trigger particular reactions in the host organism that help the pathogen survive and spread [4].

The word “pathobiont” refers to microbiota components that are often benign but may occasionally be harmful. Pathobionts are unlike “invading pathogens” that include parasites and are obtained through an external source (the environment or another host, for example) [4]. Pathobionts are opportunistic bacteria that arise when the normal microbiome is disturbed by a variety of complicated interactions involving microbial, genetic, and host variables that promote their selection and growth [5].

Pathogen infections, especially parasitic infections, can range in severity from quite innocuous to fatal. The course of the disease and the extent of damage during infection can be influenced by elements of host biology, the surrounding biotic or abiotic environment, and pathogen biology, including transmissibility, infective dose, and facultative versus obligatory behavior [1,4].

### 1.2. Microbiota and Microbiome

Communities of microbiota are constituted of bacteria, fungi, viruses, and protists taxa, and are recognized as a “hidden organ”. The genetic information of the microbiota exceeds the entire human genome, which develops steadily after birth and becomes mature near the age of three years, by 150 times [6,7].

There are some distinctions between the terms microbiota and microbiome, despite the fact that they are sometimes used interchangeably [7].

The live microorganisms found in a particular habitat, such as the oral and intestinal microbiota, are referred to as microbiota. Whereas the collection of genomes from all the environmental microorganisms is referred to as the microbiome. This includes the microbial population as well as metabolites, structural components, and environmental factors. This is where the microbiome differs from the microbiota, in that it covers a wider range [7,8].

### 1.3. Microbiota Sites and Composition

Every component of the human body that has an opening to the outside world and every surface that is exposed to it has a microbiome. The microbiota’s makeup might differ between individuals as well as between sites. This difference could be attributed to a variety of factors, including our contemporary lifestyle (which includes psychological and physical stress), diet, diabetes, allergies, the immune system, drug use, and other illnesses [9].

The gut microbiota is thought to be the most important for preserving human health [10]. It performs a number of functions including vitamin synthesis, pathogen defense, food fermentation, and immune response stimulation [11]. In addition to this, microbiota can be found in other sites including the skin, lungs, vagina, and oral cavity. The oral microbiota is thought to be the second-largest human microbial population [12].

This narrative review offers a summary of the available literature regarding the topic, focusing on parasites (helminths and protozoa) and gut-dwelling microbiota interactions, dysbiosis-associated parasitic diseases, and probiotics as a biological tool in parasitic diseases. Intestinal microbiota and gut-dwelling parasites may interact with each other and with the host immunity to alleviate or exacerbate disease. These interactions can alter the pathogenicity of both parasites and microbiota, thereby changing the infection outcomes and the overall disease profile. Parasites and microbiota interactions occur via several mechanisms, including physical alteration in both the gastrointestinal microenvironment and the adaptive and innate immune responses.

## 2. Parasites and Microbiota Interactions

Billions of people are infected with various parasites worldwide. Parasitic infections are commonly found in developing countries where they constitute an important public health problem [13]. Worldwide, infections with helminths display an imminent threat to public health, particularly in developing nations where socioeconomic factors often result in inadequate hygiene conditions [9]. Both developed and developing nations frequently have intestinal protozoa. Approximately one-third of the global population carries at least one of these protozoa, the most prevalent of which are *Entamoeba histolytica* (*E*. *histolytica*), *Toxoplasma gondii* (*T*. *gondii*), *Giardia* (*G.*) *intestinalis*, *Cryptosporidium* species (sp.) and *Blastocystis* sp. [9].

When the parasite settles in the human body, it will inevitably come into contact with and interact with members of the microbiota, since it needs a host to survive [9]. The parasites and microbiota interactions can significantly alter the gut’s physical and immune environment, paving the road for both of them to interact. These interactions can alter the pathogenicity of both parasites and microbiota, thereby changing the infection outcomes and the overall disease profile [14].

For instance, microbiota can hinder successful gut colonization by the parasite, besides distressing its replication and virulence. In turn, this results in the clinical presentation variability from asymptomatic infection to chronic parasitic disease [14]. Conversely, a parasite infection can change the way a host interacts with its microbiota either causing dysbiosis, which is characterized as a change in the composition of the microbiota, or shielding the host from it. In dysbiosis, minor microbes overgrow beneficial bacteria, increasing the severity of the pathology caused by the parasite and disrupting symbiosis [15]. Dysbiosis not only affects the pathogenicity of parasites, but also can result in several diseases, such as diabetes, cardiovascular disorders, autoimmune disorders, depression, and obesity. Therefore, a person’s gut health can reveal information about their general health.

## 3. Mechanisms of Gut Parasites and Microbiota Interactions

**Figure 1:**
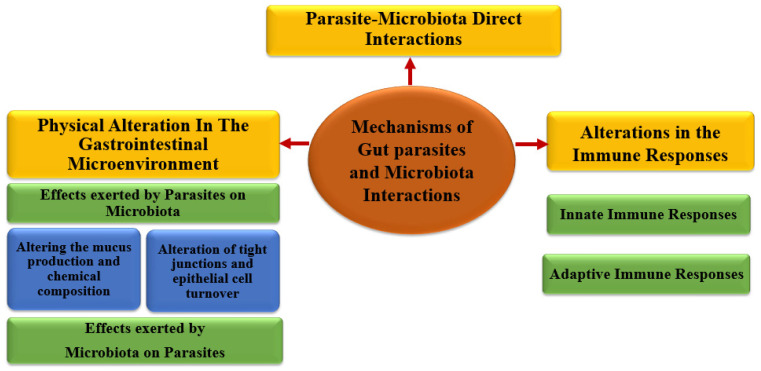
Mechanisms displaying gut parasites and microbiota interactions.

The mechanisms that display interactions of microbiota and gut parasites are demonstrated in (Figure 1) and include the following:

### 3.1. Physical Alteration in the Gastrointestinal Microenvironment

**Figure 2:**
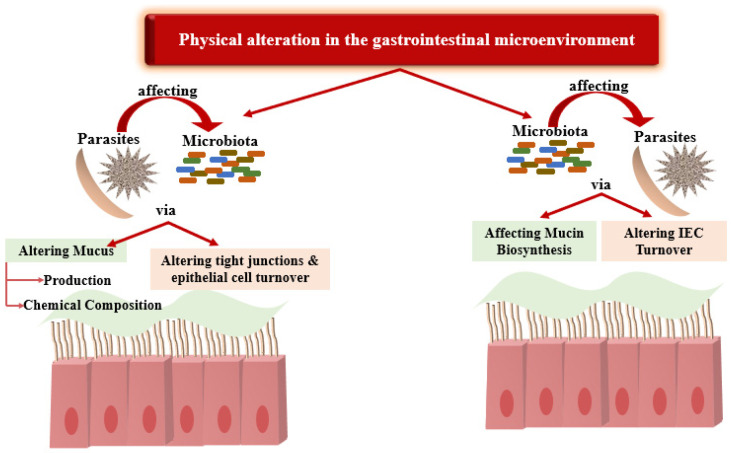
Physical alteration in the gastrointestinal microenvironment.

Colonization of the gut by parasites re-engineers the ecosystem for gut microbiota where they reside. Parasitic infections of the intestinal tract can modify the function of the epithelial barrier by affecting the chemical composition and production of mucus, altering tight junctions, and increasing the turnover of the epithelial cells (Figure 2).

#### 3.1.1. Effects Exerted by Parasites on the Microbiota

- Altering the mucus production and chemical composition

The mucus layer, which protects the host from pathogenic organisms and enables host interactions with all luminal organisms, is greatly impacted by parasites.

Several helminth infections enhance mucus production as a result of a T helper cell type 2 (Th2) immunological response brought on by interleukin (IL)-13 and IL-22, which cause goblet cell proliferation and hyperplasia [16]. Additionally, many nematodes can cause structural alterations to mucin, which is a glycoprotein that serves as the mucus base. For instance, mice that are able to expel *Trichuris muris* (*T. muris*) worms exhibit structural modification in their colonic mucin. Either increasing the production or altering the chemical composition of mucus is thought to be a host reaction that facilitates the expulsion of helminths [14,17]. On the other hand, some helminths themselves express molecules resembling mucins, which may be involved in immune evasion and host-cell adhesion [14].

In the same way, the quantity and structure of intestinal mucus are also altered by parasitic protozoa. During pathogenesis, certain species, such as *Tritrichomonas suis*, *G*. *intestinalis*, and *E*. *histolytica*, are able to pass the mucus barrier due to the production of mucolytic enzymes [18].

In addition to generally increasing goblet cell numbers, *T*. *gondii* promotes a change in mucus production towards neutral and more acidic mucins, which is expected to facilitate the expulsion of parasites and increase mucus fluidity [19].

These changes induced by the parasite in the mucus layer could be either harmful or beneficial to the microbiota balance. The inhibitory impacts of parasites on microbiota may be exerted by altering the microbiota’s attachment to the epithelial surface, changing its accessibility to nutrients, in addition to causing disorders in its evacuation out of the gut [14]. Nevertheless, the parasite-induced increase in mucus production might upgrade the abundance of some species of microbiota. For instance, species like *Verrucomicrobia*, *Firmicutes*, *Bacteroidetes*, and *Actinobacteria* consume carbohydrates from mucus as a source of carbon. Additionally, the parasite-induced alteration in the chemical composition of mucus is known to enhance the growth of mucus-utilizing genera like Clostridiales [20].

- Alteration of tight junctions and epithelial cell turnover

Gut parasites like helminths increase the turnover of the epithelial monolayer, causing potentially debilitating effects on the gut-dwelling microbes. Microbiota with high replicating rates increases selectively to evade being evacuated from the gut. Also, the pathogenesis of protozoa may evolve parasite attachment, the distraction of tight junctions, cell invasion, or epithelial cell damage. Therefore, gut microbes come into significant contact with the epithelial barrier, or even translocate across it, as in the case of toxoplasmosis, cryptosporidiosis, and giardiasis infections [21].

#### 3.1.2. Effects Exerted by Microbiota on Parasites

Microbiota affects mucin biosynthesis at the molecular level, which in turn affects the colonization, persistence, and fertility of several parasites. Also, gut microbiota alters the turnover of the intestinal epithelial cells (IEC). For example, Gram-positive bacteria mediate the repair in the gastrointestinal tract and increase the turnover of the IEC, i.e., “epithelial escalator”, thus expelling nematodes and hindering their colonization [22].

### 3.2. Alterations in Adaptive and Innate Immune Responses

Both microbes and parasites can figure out the gastrointestinal tract immune context, allowing it to induce selective growth of microbial species.

#### 3.2.1. Innate Immune Responses

Helminths can affect the innate immune reactions to the microbiota by modulating the sensitivity and expression of receptors called toll-like receptors (TLRs), which can impact the host defense against bacterial infections. Moreover, helminths alter the production of small peptides, called antimicrobial peptides (AMPs) that have an effect on bacteria, fungi, and viruses, by the host. Because host TLR responses and AMPs have definite microbicidal effects, they can impact gut microbiota composition, causing dysbiosis [23].

Microbiota–parasite interactions may also evolve activation of the inflammasome, which alters the microbiota, causing dysbiosis. Conversely, triggering the inflammasome by the microbiota induces a pro-inflammatory context efficient to eliminate protozoa [23].

#### 3.2.2. Adaptive Immune Responses

Several intestinal nematodes and gut microbiota, e.g., *Lactobacillus* sp., *Bacteroides fragilis*, *Bifidobacterium infantis*, and *Clostridium* sp. stimulate regulatory T cells (Treg cells), and so these organisms may promote a tolerogenic ecosystem that withstands their persistence and proliferation. Herein, parasites and microbiota synergize with each other, yet the distraction to either community may disturb tolerance and result in disease [24].

### 3.3. Parasite-Microbiota Direct Interactions

This type of interaction does not involve the host and is caused chiefly by the prolonged coexistence of both parasites and microbiota in the gut. Some helminths depend on microbial composition to develop inside their host. Interestingly, germ-free animals were found to be resistant to colonization with some helminthic infections, e.g., *Heligmosomoides polygyrus* (*H. polygyrus*), *Trichinella spiralis* (*T. spiralis*), and *Nippostrongylus brasiliensis* [14].

Parasite–microbiota direct interactions are also obvious among protozoa. For instance, *E*. *histolytica* phagocytoses enteropathogenic bacteria like *Escherichia coli* and *Shigella dysenteriae*, which in turn prompts virulence and invasion of *Entamoeba* in the host epithelium, causing dysentery [25].

Several helminths also produce helminth defense molecules (HDMs), peptides that are similar to the antimicrobial peptides of humans. HDMs have direct bactericidal properties and immunomodulatory impacts by modifying host immune reactions, therefore enhancing their colonization and parasitism inside the host [26].

Finally, direct interaction may involve the possible overlapping of the available nutrient resources, either by competition for a single nutrient or cross-feeding where one species consumes the excretory byproducts of another. In other instances, direct interactions involve the predator-prey relationship between eukaryotic parasites and prokaryotic microbiota where a helminth or a protozoa prey on enteric bacteria as a source of nutrients [14].

## 4. Parasite and Microbiota Interaction’s Implications on Health and Disease of the Host

**Figure 3:**
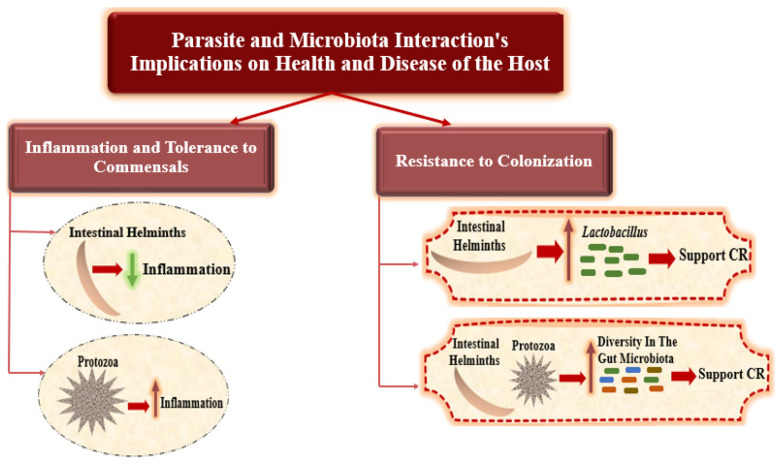
Parasite and microbiota interaction’s implications on health and disease of the host.

Disruptions affecting the parasite and microbial ecosystems of the gut induce numerous health disorders. New understandings of the basic processes driving interactions between microbiota and parasites might eventually enable the prediction of the effects of changes to the gut community on host health, as well as a better understanding of the diverse outcomes of parasitic infection [14]. Several studies have shown how interactions between microbiota and parasites affect health through a variety of pathways (Figure 3).

### 4.1. Inflammation and Tolerance to Commensals

Numerous parasites, including protozoa and helminths have a significant impact on intestinal inflammation, either through enhancement or reduction.

#### 4.1.1. Parasitic Helminths as Inflammatory Reducers

Interestingly, many inflammatory diseases are reported to improve with helminth infections. Numerous investigations have shown that helminths’ potential to prevent inflammatory diseases could be somewhat mediated by their impacts on the microbiota [14,16]. A number of nematodes enhance short-chain fatty acid- (SCFAs) producing gut bacteria. SCFAs are byproducts of bacterial fermentation that travel throughout the body, regulating systemic immune responses by triggering Treg cells [27].

The potential of helminth-based treatments for the management of disease has been brought to light by several recent studies [28]. Numerous studies have documented improvements in ulcerative colitis and Crohn’s disease clinical manifestations among patients who received oral *Trichuris suis* (*T. suis*) ova [29]. The nematode *H. polygyrus* guards from airway inflammation in the asthma model in mice; this was at least partly influenced by modifications to the microbiota and the rise in cecal SCFA concentrations [30]. It has even been reported that allergic asthma is prevented by transferring the microbiota from mice infected with *H. polygyrus* [9,28,31]. Additionally, another study documented that *H. polygyrus* promoted changes in the colon’s epithelial barrier function and dramatically lowered colitis-associated inflammation in mice. *H. polygyrus* stimulates *Lactobacillus* species growth, which is linked to better gut health, inflammation management, and the induction of tolerogenic immunological responses [9,28,31]. *Necator americanus* in humans and *Ascaris suum* in pigs were two other nematode species that led to raised SCFA gut levels, indicating that SCFA elevations may be a frequent sign of helminth infection [30]. The hygiene hypothesis supports the idea that helminths protect against inflammatory illnesses, linking the rise in allergic and autoimmune diseases in developed countries to reduced exposure to gastrointestinal helminths and commensal microorganisms [28,29]. Another theory stated that the loss of old friends’ microorganisms heightens the sterile, aging-associated inflammation called inflammaging, which is connected to several age-related diseases such as cancer, dementia, and cardiovascular disease. Recently, treatment with a parasitic nematode’s secreted glycoprotein guarded against aging in a mouse model by enhancement of anti-inflammatory mechanisms [29,32].

#### 4.1.2. Parasitic Protozoa as Inflammatory Enhancers

Compared to helminths, a number of protozoa have the ability to induce or worsen inflammatory bowel disease. Once again, associations with the gut microbiota are involved [33,34]. Several parasitic protozoa can endure for extended periods of time in the intestinal lumen without causing any noticeable damage. Usually, pathogenesis includes epithelium adherence and occasionally invading the epithelium, which induces inflammatory immunological reactions [14].

By enabling the translocation of commensals that are often tolerated over the epithelial barrier, these virulence-associated mechanisms can exacerbate inflammatory illness. For instance, post-infectious irritable bowel syndrome (IBS), which is brought on by *G. lamblia*, has been linked to alterations in host-commensal interactions induced by parasites. According to recent research, a *Giardia* infection can lead to post-infectious IBS by causing bacterial penetration, mucosal inflammation, and chronic tight junctional damage that lasts long after the parasite is removed [35,36]. It has long been recognized that *T. gondii*-induced ileitis is mediated by the microbiota. Mice exposed to high doses of infection develop ileal inflammation as a result of pathobionts that are adhesive and invasive (like *Escherichia coli* and Bacteroides/Prevotella), invading the mucosa and altering the composition of gut microbiota, with Gram-positive bacteria switching to Gram-negative bacteria and reducing diversity [37,38].

### 4.2. Resistance to Colonization

Colonization resistance (CR) is defined as the process by which host intestines are shielded by the microbiota from exogenous pathogens [14]. Until recently, it was thought that parasites living in the gut can modify susceptibility to bacterial infection in the gut through mechanisms not involving the microbiota. For instance, helminths can combat bacterial infections like *Salmonella enterica* Serovar Typhimurium, which thrive in an inflammatory environment by promoting an anti-inflammatory gut environment, [39] or, on the other hand, encouraging pathogens that inflammation might otherwise be able to efficiently control [40]. However, it is plausible that the influence of parasites on the gut microbiota and interactions between microbes mediates, at least in part, their effects on gut bacterial infections.

Numerous intestinal helminths are known to encourage the growth of *Lactobacillus* sp., which are frequently regarded as probiotics and have been demonstrated to support CR [41,42]. For instance, *Lactobacillus delbrueckii* lessens *Clostridium difficile*’s cytotoxicity and its adhesion to colonic cells in vitro [43]. Therefore, it makes sense that helminth infection might improve CR by encouraging *Lactobacillus* growth.

Additionally, gut microbial diversity can be changed by parasite infection, which is documented in several studies. The destruction of CR and the causes of inflammatory illnesses are likely to be significantly influenced by reduced microbiota diversity, hence such impacts may have additive effects on pathogen susceptibility.

It’s interesting to note that a number of investigations on humans showed increased diversity in the gut microbiota of those with intestinal helminth [44,45] or protozoa infections [46,47], or that this diversity changed after parasite therapy [48]. In conclusion, parasites may have an impact on the variety and composition of the microbiota, which may have an impact on CR.

## 5. Microbiota Modulations in Relation to Parasitic Infections

Modulating the microbiota can improve the treatment of parasitic infections and dysbiosis by understanding the mechanisms and consequences of interactions between intestinal parasites and microbiota.

### 5.1. Utilizing Probiotics and Prebiotics to Treat Parasitic Infections

With over a billion cases of parasitic infections worldwide, in spite of years of strict control, there is no reliable human vaccination to prevent them, and parasites represent a major health concern [49,50]. In addition, a pervasive drug resistance issue has been brought about by the broad use of existing chemotherapeutic drugs against parasites [49,50]. To cope with this problematic restriction of antiparasitic medications, various alternative therapeutics based on probiotics or prebiotics have been developed.

#### 5.1.1. Probiotics in the Treatment of Parasitic Infections

Probiotics are outlined by WHO as live microbes that, when given in sufficient quantities, can help a host’s health [49]. Most of them are gram-positive bacteria that have been obtained from the human gut microbiota or from different dairy-related products, including kulfi (a dairy-frozen dessert from the Indian subcontinent), lassi (a flavorful drink that occasionally contains cumin), and curds. They have non-pathogenic properties and are resistant to acidity and low pH. They come in meal, tablet, or powder form and can be purchased from pharmacies as well as internet retailers. The most popularly used probiotics are *Lactobacillus*, *Enterococcus*, *Bifidobacterium*, yeast, and some fungi [2,50].

The minimum amount required for the ingested probiotics to have the survival capacity to outcompete the local bacteria in the gastrointestinal tract is five days, with five billion colony-forming units (CFU) ingested per day. This is crucial for achieving the beneficial effects of probiotics [50].

Probiotics act through several mechanisms. These mechanisms include aiding the colonization and normalization of disturbed gut microbes, excluding pathogens through competitive exclusion and bacteriocin production, modifying enzyme activities involved in metabolizing carcinogens and toxins, and producing volatile fatty acids essential for energy homeostasis and peripheral tissue function [51].

Additionally, probiotics support the formation of mucin and intestinal barrier integrity, while regulating the immune system’s and gut-associated lymphoid tissue’s activity [51].

In the same way, probiotics may disrupt the physiology of intestinal parasites. Probiotics may also be a crucial component of parasitic infection control measures, since their products possibly have antiparasitic activities, and can diminish many parasites’ virulence [50]. So, probiotics can be introduced as antiparasitic therapy or added to conventional antiparasitic medications.

Despite the fact that studies examining the impacts of probiotics on helminth parasitic infections have inconsistent outcomes, most published research has reported a decline in helminth loads when using probiotic treatment. This shows that some species of probiotics, such as *Bifidobacterium* and *Lactobacillus* species, may be able to defend against helminth infection by regulating the host immune system, creating antimicrobial peptides, and competing with pathogens for resources and space [49].

According to several studies, *Lactobacillus* may provide protection by triggering a potent Th2 response [52], or helminth-specific antibodies [53].

In the same way, it has been demonstrated that a number of *Lactobacillus* strains prevent protozoal parasitic infection, through enhancing the humoral immune response towards *Giardia,* and decreasing the trophozoite’s adhesion to the mucosal surface [54].

Probiotics have been shown to have beneficial effects against several parasites, whether taken alone or in combination, as listed in Table 1.

#### 5.1.2. Prebiotics in the Treatment of Parasitic Infections

Prebiotics are defined as dietary substrates that improve the host’s health by promoting the proliferation or actions of particular gut microbes [2,14].

With enough knowledge of the interactions between the parasites and microbiota, as well as the impact of diet on bacteria, dietary prebiotics could be used as an alternative to probiotics in the treatment of parasites. Prebiotics may be formulated to promote the proliferation of particular microorganisms in the gut that suppress parasites or lessen their pathogenicity. In this way, modifications of diet alone may be a successful intervention against parasitic infection [14].

The original purpose of prebiotics was to investigate the stimulating properties of probiotics. Prebiotics that are well-known include lactulose, inulin, galacto-oligosaccharides (GOS), and fructo-oligosaccharides (FOS). Prebiotics are primarily employed to regulate the lactic acid and acetate-producing *Bifidobacterium* and *Lactobacillus* strains, and also to preserve the host’s health through prebiotic fermentation [71].

Several researchers demonstrated that consuming prebiotics can promote the selective enrichment of digestive tract probiotics, which in turn controls the immune system and wards against infections [72].

Numerous studies imply that this approach is feasible. For example, it has been demonstrated that adding a complex polysaccharide, metabolized by a specific set of microorganisms known as inulin, to pigs’ diets significantly lowers their susceptibility to helminth infections caused by *Trichiuris suis* (*T. suis*) and *Oesophagostomum dentatum* [73]. Nonetheless, these results suggest that prebiotics may be a useful strategy for lowering parasite susceptibility by modifying the gut microbial population.

While a convincing body of evidence exists on the effectiveness of certain probiotics and prebiotics for a broad range of health applications, their consistent implementation into nutrition and healthcare remains limited [74]. Their usefulness may be restricted by a number of ongoing issues. A better utilization of probiotics and prebiotics in relation to disease will likely result from an understanding of the current challenges [75].

Probiotic and prebiotic efficacy are thought to be primarily influenced by variations in dose type and host. Crucially, the desired outcome will not be realized if probiotics cannot reach the intended location. Bacteriocins and probiotics that produce them can preserve microecological balance and gut health, while issues like large-scale manufacture and instability in specific conditions restrict their future use [7,75].

### 5.2. Anti-Parasitic and Antibiotic Treatments’ Effects on the Interactions Between Parasites and Microbiota

A deeper understanding of parasites and microbiota interactions will be also fundamental in anticipating the broader health effects of particular anti-parasitic and antibiotic treatments.

#### 5.2.1. Anti-Parasitic Treatments’ Effects on Microbiota

According to experimental research, anthelmintic therapies may change a host’s vulnerability to other bacterial and protozoan infections [76]. This could be because anthelmintic therapies eliminate the immunomodulatory effects of helminths or their competitive relationships with other species.

Anti-parasitic treatments can also impact gut microbiota. For example, anthelmintic therapy reversed the compositional changes and reduced microbiota diversity observed in mice experimentally infected with *T. muris* [77]. Similarly, when albendazole was administered to humans with helminth infections, the overall diversity of the microbiota declined, with a reduction in Clostridiales and upregulation in Bacteroidales [45].

#### 5.2.2. Antibiotic Treatments’ Effects on Parasites

Similarly, antibiotic treatment can alter a host’s vulnerability to parasitic illnesses by affecting interactions between parasites and microbes. For instance, giving antibiotics to mice lowers the amount of both aerobic and anaerobic bacteria in the gut, as well as the quantity of *T. muris* eggs that hatch and the ensuing worm loads [78].

It has been demonstrated that certain bacteria hinder the growth of *Cryptosporidium*, *Giardia*, and *Eimeria*. So, administration of antibiotics increases the parasite burden and makes the host prone to this infection [14].

The relationship between *Blastocystis* colonization and the composition of gut microbiota is believed to be affected by the use of antibiotics. According to a human population study, the group taking antibiotics had a much lower prevalence of *Blastocystis*. This could be explained as an indirect effect of antibiotic therapy, which includes the suppression or elimination of bacterial populations that *Blastocystis* may need for colonization, not a direct destructive effect of antibiotics on *Blastocystis* [79].

## 6. Conclusions

Parasites and microbiota interactions can significantly alter the gut’s physical and immunological environment, paving the road for both of them to interact. These interactions can change the pathogenicity of both parasite and microbiota, therefore altering the outcome of the infection and affecting the overall nature of the disease. New understandings of the basic processes driving interactions between microbiota and parasites might eventually enable the prediction of the effects of changes to the gut community on host health, as well as a better understanding of the diverse outcomes of parasitic infection. Through understanding the mechanisms and effects of the interactions between intestinal parasites and the microbiota, treatments for parasitic infections and microbiota dysbiosis may be improved. Probiotics are one of the new biological therapy options that can be introduced, especially in light of the growth of medication resistance and side effects.

## Figures and Tables

**Table 1:** Effects of Probiotics on some parasites.

Parasite	Host	Used Probiotic	Dose/Route	Mechanisms	Efficacy	Ref.
Helminthic infections						
*Schistosoma mansoni*	Mice	*Lactobacillus* (*L*.) *acidophilus* (ATCC 4356) and *L. delbrueckii* subsp. *bulgaricus* (DSM 20080) mixture	Orally given 100 μL of probiotics solution either 1 week before or on the day of infection	Decreased MMP-9 hepatic levels, lipid peroxidation, and increased levels of reduced glutathione	Adult worm reduction by 68 for pre- and 60% for post-infection	[55]
	Mice	*Lactobacillus sporogenes*	Orally given from the first day of infection, 12.5 million spores/mouse/week for 8 weeks	Decreased DNA damage; lessens intestinal and liver damage	Decreased egg and worm counts	[56]
	Mice	*Zymomonas* (*Z*.) *mobilis*	Orally administered 10^9^ CFU/mL culture of *Z. mobilis* at 0.3 mL/day for 7 days before or 7 days after the infection	*Z. mobilis* enhanced immune response against *S. mansoni*	Adult worm reduction by 24% in prophylactic and 61% in curative group	[57]
*Echinococcus granulosus*	Mice	*Acidophilus* plus probiotic which is *L. acidophilus*, *L. casei*, and *L*. *rhamnosus* mixture	Intraperitoneally, probiotic dilutions of 9 × 10^6^/0.1 mL and 30 × 10^3^/0.1 mL CFU were administered before and after infections.	Stimulation of immune response	Decrease in cysts’ diameter, weighting, and digit, and result in 98.03% hydatid cysts reduction 6 months post-infection	[58]
*Ascaris* and *Parascaris* sp.	Both in vitro and in vivo in pigs, mice and foals	A deactivated bacterium (para probiotic) that expresses *Bacillus thuringiensis* Cry5B named IBaCC	Orally given IBaCC containing Cry5B at a dose of 20 mg/kg body for mice, 30 mg/kg for pigs, and for foals through a nasogastric tube at 30 mg/kg	An effect on parasites directly and is independent of a healthy immune response	IBaCC induced toxicity to *A. suum* larvae in vitro, with a high efficacy against *A. suum* in mice. One dosage of Cry5B IBaCC almost eradicated *A. suum* in pigs and decreased the number of fecal eggs in parascaris-infected foals to zero	[59]
*Trichinella spiralis*	Mice	Wild type and IL-4 recombinant *L. plantarum* NC8 was used	Orally given to mice 1 × 10^9^ CFU of bacterial culture of the recombinant NC8-pSIP409-pgsA-mIL-4 and *L. plantarum* NC8 before challenge with *T. spiralis*	Enhance intestinal mucosal immunity and accelerate adult worm eliminationby increasing IgG1 serum levels and mucosal secretory IgA and upregulating Th2 immune response	*Lactobacillus* sp. can partially decrease parasite load with a stronger effect with IL 4	[60]
Protozoal infections						
*Cryptosporidium parvum*	Human	*L. rhamnosus* GG and *L. casei* shirota	10^9^ units/day of *L. rhamnosus* GG and 6.5 × 10^9^ units/day of *L. casei* shirota for four weeks started after infection	Reduction of theparasite burden in the intestinal epithelium	Clinical case resolution	[61]
	Calf	*Bacillus* (*B*.) *brevis*, *Pseudomonas* (*P*.) *alcaligenes* and *Enterococcus* (*E*.) *faecium*	Concomitant administration & infection	-	Insignificant effect	[62]
	Cell culture	*E.faecium*, *B.brevis*, and *P. alcaligenes*	Oocysts were inoculated into BCFs at 3 × 10^6^/mL and incubated for 2 weeks at 37 °C	Premature excystation of oocyst	75–100% reduction	[63]
	Mice	*L. reuteri* 4000, 4020	Orally fed day-by-day for 4 weeks	Secretion of unidentified antimicrobial products	50–75% oocyst shedding reduction	[64]
*Giardia lamblia*	Both In vitro and in vivo in mice	Bacteriocins produced from recently isolated strains of *L. acidophilus* (P106) and *L. plantarum* (P164) in Egypt	50 µg of *L. acidophilus* bacteriocin for in vitro culture. Orally given for 5 successive days of 50 µg/mouse *L. acidophilus* bacteriocin	Bacteriocin reduced trophozoites adherence	Bacteriocin reduced trophozoites number by 58.5% invitro and reduced parasite load, and improved intestinal pathology of infected mice in vivo	[65]
	Mice	*L. acidophilus*, *L. helveticus*, and *Bifidobacterium bifdum*	A bacterial mixture containing 1 billion CFU/1 capsule before and after infection	Inhibited the growth of the parasite, enhanced antioxidant capacity, decreased oxidants, boosted cellular and humoral immunity, and adjusted the intestinal mucosa’s inflammatory state.	Preventive effects when administered before infection and significantly decreased infection severity (87.5% after 20 days) and intestinal alterations in the therapeutic group	[66]
	Human	*Saccharomyces* (*S.*) *boulardii*	For ten days, administration of 250 mg twice per day of *S. boulardii* capsules together with 750 mg of metronidazole three times a day	This combination boosted intestinal enzymes, triggered immunological responses, raised intestinal disaccharidases, and produced an intestine trophic effect.	Beneficial anti-giardial effects when used as an adjuvant treatment in adult patients	[67]
*Entamoeba histolytica*	Human	*Saccharomyces boulardii*	Oral Treatment of acute diarrheal patients with either antibiotics alone or combined with three daily doses of 250 mg of *S. boulardii* for ten days.	The yeast is very good at restoring the positive benefits of a healthy gut flora.	Combined therapy after four weeks significantly decreased the symptoms’ duration and excreted cyst number	[68]
*Toxoplasma gondii*	Mice	*L. delbrueckii* and *L. fermentum*	*Lactobacillus* 1 billion per day either alone or combined 100 mg/kg of sulfamethoxazole-trimethoprim for 14 days, 7 days prior to and 7 days following infection	Immunological impact of probiotics through strengthening the host’s defenses against infection	With an 82% decrease in tachyzoites, combined therapy boosted survival rate and time to 95% and 16 days, respectively	[69]
*Plasmodium chabaudi*	Mice	*L. casei* ATCC7469	7–15 days before infection	*L. casei* induced nonspecific protection against *P. chabaudi*	Parasitemia reduction by 25–50%	[70]

## Data Availability

All datasets analyzed or generated during the study are included in this published version.

## Abbreviations

AMPs: Antimicrobial Peptides; B.: Bacillus spp.; BCFs: Bacterial Cell-Free Filtrates; CFU: Colony Forming Units; Cry5B: Crystal (Cry) protein; E.: *Enterococcus* spp.; *E. histolytica*: *Entamoeba histolytica*; *G.*: *Giardia*; *H. polygyrus*: *Heligmosomoides polygyrus*; HDMs: Helminth Defense Molecules; IBaCC: Inactivated Bacterium with Cytosolic Crystal; IBS: Irritable Bowel Syndrome; IEC: Intestinal Epithelial Cells; IL: Interleukin; *L*.: *Lactobacillus*; MMP-9: Matrix Metalloproteinases 9; NLRP: Nod-Like Receptor-Pyrin Domain Containing; NO: Nitric oxide; *P*.: *Pseudomonas* spp.; *S*.: *Saccharomyces*; SCFAs: Short-chain fatty acids; sp.: Species; *T. gondii*: *Toxoplasma gondii*; *T. muris*: *Trichuris muris*; *T. suis*: *Trichuris suis*; *T. spiralis*: *Trichinella spiralis*; Th2: T helper cell type 2; TLRs: Toll-Like Receptors; Treg cells: Tolerogenic T cells; WHO: World Health Organization; *Z. mobilis*: *Zymomonas mobilis*.
